# Plasma Membrane Abundance of Human Aquaporin 5 Is Dynamically Regulated by Multiple Pathways

**DOI:** 10.1371/journal.pone.0143027

**Published:** 2015-11-16

**Authors:** Philip Kitchen, Fredrik Öberg, Jennie Sjöhamn, Kristina Hedfalk, Roslyn M. Bill, Alex C. Conner, Matthew T. Conner, Susanna Törnroth-Horsefield

**Affiliations:** 1 Molecular Organization and Assembly in Cells Doctoral Training Centre, University of Warwick, Coventry, United Kingdom; 2 Department of Chemistry, University of Gothenburg, Gothenburg, Sweden; 3 School of Life and Health Sciences, Aston University, Birmingham, United Kingdom; 4 Clinical and Experimental Medicine, The Medical School, University of Birmingham, Birmingham, United Kingdom; 5 Biomedical Research Centre, Sheffield Hallam University, Sheffield, United Kingdom; 6 Department of Biochemistry and Structural Biology, Centre for Molecular Protein Science, Lund University, Lund, Sweden; University of Bari Aldo Moro, ITALY

## Abstract

Aquaporin membrane protein channels mediate cellular water flow. Human aquaporin 5 (AQP5) is highly expressed in the respiratory system and secretory glands where it facilitates the osmotically-driven generation of pulmonary secretions, saliva, sweat and tears. Dysfunctional trafficking of AQP5 has been implicated in several human disease states, including Sjögren’s syndrome, bronchitis and cystic fibrosis. In order to investigate how the plasma membrane expression levels of AQP5 are regulated, we studied real-time translocation of GFP-tagged AQP5 in HEK293 cells. We show that AQP5 plasma membrane abundance in transfected HEK293 cells is rapidly and reversibly regulated by at least three independent mechanisms involving phosphorylation at Ser156, protein kinase A activity and extracellular tonicity. The crystal structure of a Ser156 phosphomimetic mutant indicates that its involvement in regulating AQP5 membrane abundance is not mediated by a conformational change of the carboxy-terminus. We suggest that together these pathways regulate cellular water flow.

## Introduction

The flux of water across biological membranes is facilitated by transmembrane protein channels called aquaporins (AQPs). AQPs passively transport water in response to osmotic gradients, while excluding the movement of ions and protons [1] and thus are important for cell volume regulation [2]. In humans, thirteen members of the AQP family (AQP0-12), with subtle functional differences, are expressed with different tissue-specific and time-dependent profiles [3].

Eukaryotes have evolved to fine-tune water transport through AQPs by three main regulatory mechanisms: (i) at the transcriptional/translational level; (ii) by conformational change or “gating” and (iii) by translocation to the membrane in response to a trigger. Regulation by AQP gene expression and/or AQP protein degradation can be achieved over a timescale from hours to days. However, this does not account for the dynamic control of AQPs that may be necessary to rapidly alter membrane water permeability in response to environmental or cellular signals. Instead, this can be achieved by gating; a conformational change of the AQP protein that alters the permeability of the pore. In addition, translocation can regulate the number of AQP molecules present in the target membrane, altering membrane water permeability by changing the number of pores present.

Structures of gated AQPs have revealed the molecular details of AQP gating by phosphorylation, pH and Ca^2+^ for the spinach aquaporin SoPIP2;1 [4] and mechanosensitivity for the yeast aquaporin AQY1 [5]. Furthermore, mammalian AQP0 is suggested to be gated in a pH and Ca^2+^-dependent manner, the latter being mediated by an interaction with calmodulin, as described by a recent structural model [6]. While gating of other mammalian AQPs remains to be conclusively shown, translocation is a common regulatory mechanism. The best-characterised example of this type of regulation is that of human AQP2 in the kidney: AQP2 abundance in the apical membrane is dependent on vasopressin-activated phosphorylation of a carboxy-terminal serine residue (Ser 256) by cAMP-dependent protein kinase A (PKA) [7]. Phosphorylation in response to a hormonal trigger has also been shown to mediate membrane translocation of AQP1 [8], AQP5 [9–11] and AQP8 [12], on a timescale of minutes to hours. Translocation in response to an osmotic stimulus has been demonstrated to regulate AQP1 activity on a timescale of seconds; exposure to hypotonic conditions resulted in rapid recruitment to the cell surface via a mechanism dependent on transient receptor potential channels, extracellular calcium influx, calmodulin, and the phosphorylation of two threonine residues (Thr 157 and Thr 239) of AQP1 [13].

AQP5 is found in tissues such as the lungs, airways and secretory glands and consequently plays a major role in the generation of saliva, tears and pulmonary secretions [14–16]. AQP5 dysregulation has been implicated in several disease states, including bronchitis, cystic fibrosis [17] and Sjögren’s syndrome [18]. AQP5 translocation has been shown to be affected by cAMP in a PKA-dependent manner, with exposure to elevated intracellular cAMP levels causing a short-term (minutes) decrease in AQP5 membrane abundance whereas long-term (8 hours) exposure increased total AQP5 protein [15]. There are two consensus PKA sites in AQP5: Ser 156 in cytoplasmic loop D [19, 20] and Thr 259 [10] in the carboxy-terminus; the latter corresponds to Ser 256 in AQP2. AQP5 can be directly phosphorylated by PKA at Ser 156 and Thr 259 [21]. Notably, Ser 156 was phosphorylated preferentially in certain tumors suggesting that cell proliferation can be modulated by phosphorylation of this site although the constitutive membrane abundance of an S156A mutant was not distinguishable from wild-type AQP5 [22]. Based on the crystal structure of human AQP5 it was hypothesized that phosphorylation of Ser 156 could cause structural changes in loop D that would break its interaction with the carboxy-terminus, thereby flagging the protein for translocation to the plasma membrane [23].

In order to investigate the role of Ser 156 in the membrane translocation of AQP5, we used real time translocation studies in living HEK293 cells; GFP-tagged full-length AQP5 mutants were designed to either abolish or mimic phosphorylation of Ser 156. Our data show that the phosphomimetic mutation of Ser 156 to glutamate (S156E) increased constitutive membrane expression of AQP5. Inhibition of PKA increased constitutive membrane expression of wild-type, S156E and S156A, suggesting that the effect of PKA on AQP5 translocation is not solely dependent Ser 156. We further show rapid membrane translocation upon a hypotonic stimulus independently of both Ser 156 phosphorylation and PKA activity. Finally, we have used x-ray crystallography to show that the phosphomimetic S156E mutation does not cause any significant structural changes to the protein as previously suggested. We propose that three independent mechanisms regulate the membrane abundance of AQP5, one of which involves phosphorylation of Ser 156.

## Materials and Methods

### Cloning of AQP5-GFP fusion constructs for analysis in HEK293 cells

Human full-length AQP5 was fused with carboxy-terminal GFP using the Invitrogen Gateway™ cloning system according to the instructions provided by the supplier. For directional cloning of blunt-ended PCR products into an entry vector using the Gateway™ system, four bases (GGGG) were added to the 5′-end of the forward primer followed by the 25bp *att*B1 attachment sequence (underlined, below). This was followed by five bases (bold) to introduce a Kozak sequence upstream and to keep the sequence in frame with the AQP coding sequence. Finally 18-25bp of the AQP5 sequence were added to create the amino-terminal forward primers, 5′-GGGG ACA AGT TTG TAC AAA AAA GCA GGC T **CC ACC** ATG–AQP5(18-25bp)-3′. For the reverse primer, four bases (GGGG) were added to the 5′-end followed by the 25bp *att*B2 attachment sequence (underlined) and then one base (bold) was added to keep the sequence in frame with the AQP5 coding sequence. Finally 18-25bp of the AQP sequence without the stop codon were added to create the carboxy-terminal forward primers 5′-GGG GAC CAC TTT GTA CAA GAA AGC TGG GT **C**–AQP5(18-25bp)-3′. KOD polymerase was used in PCR amplification of the AQP cDNA. Samples were heated to 94°C for 2 min, followed by 30 cycles of 94°C for 30 s, 55°C for 30 s and 68°C for 3 min and then 68°C for 7 min. Purified PCR products were sub-cloned into the pDONR221™ entry vector (Invitrogen) using the *att*B1 and *att*B2 sites in a reaction with Gateway™ BP Clonase™ enzyme mix (Invitrogen). pDONR221™ vectors containing the required sequences were recombined with the pcDNA-DEST47 Gateway™ vector using the *att*L and *att*R reaction with Gateway™ LR Clonase™ enzyme mix (Invitrogen). This created expression vectors with the cycle 3 mutant of the GFP gene at the carboxy-terminus of the AQP gene of interest, which were subsequently expressed as fusion proteins. The mutant constructs S156E and S156A were amplified using the well-established, modified QuikChange procedure (Stratagene), as previously described [13] and according to their manual. All plasmids were handled and purified using standard molecular biological procedures.

### Cell culture and transfection

HEK 293 cells were routinely cultured in Dulbecco’s modified Eagle’s medium (DMEM) supplemented with 10% (v/v) fetal bovine serum in humidified 5% (v/v) CO_2_ in air at 37°C. Cells were seeded into 30 mm Fluorodish™ dishes and transfected after 24 h at 50% confluence using the Transfast (Promega) transfection protocol with 2 μg of DNA/dish. PKA inhibition was achieved by incubation with 3.6 μM myristoylated PKI 14–22 amide (Enzo Life Sciences, Exeter UK) for 30 minutes.

### Confocal microscopy

AQP5-GFP fusion proteins were visualized in live cells enclosed in a full environmental chamber by confocal laser scanning microscopy. Confocal images were acquired 24 h post-transfection with a Leica SP5 or Zeiss 780 laser scanning microscope using a 63× (1.2 NA) water immersion objective. Images were acquired using an argon laser (excitation 488 nm; emission band pass 505–530 nm) for GFP, UV excitation and a He-Ne laser (excitation 543nm; emission filter long pass (LP) 650 nm). Cells were visualized in control medium (DMEM) that had an inorganic salt concentration of 120 mM, a glucose concentration of 25 mM and an osmolality in the range 322–374 mosM/kg H_2_O. Hypotonic medium has an osmolality in the range 107–125 mosM/kg H_2_O through dilution of DMEM by a factor of 3 with water.

### Determination of sub-cellular localization

Protein localization was measured using a line profile (pixel density) traced on each transfected cell. Localization data are representative of three to five cells from at least three independent experiments. Line expression profiles were generated and analyzed with the program ImageJ (http://rsb.info.nih.gov/ij/) and are indicated in yellow and displayed beside each confocal image. A minimum of five line profiles were measured, distributed at regular intervals covering the plasma membrane and the cytosol, but avoiding the nucleus of a minimum of three cells from at least three independent experiments The fluorescence intensity over this distance was also measured and the difference between the peak and the plateau of fluorescence was divided by the maximum fluorescence along the line scan to calculate the percentage of fluorescence at the membrane. This was termed the relative membrane expression (RME) [24, 25]. The overlay of the GFP image with the bright-field image indicated integration of GFP-tagged AQP5 at the plasma membrane as well as in the cytoplasm of HEK293 cells. In addition, correct membrane insertion of AQP5-GFP and both S156 mutants was suggested by colocalization with the fluorescent plasma membrane marker FM 4–64 as previously described for AQP4-GFP..

RME values were compared by one way ANOVA followed by either paired (for images of the same cell before and after treatment) on unpaired (for images of different cells under different conditions) post-hoc t-tests. The p values from the post-hoc tests were multiplied by the number of pairwise comparisons (Bonferroni correction); p < 0.05 after correction was considered statistically significant.

### Cloning of hAQP5 S156E for structural analysis

A single mutation of Ser 156 to glutamate was introduced into a vector encoding wild-type human AQP5 using the QuikChange^®^ Site-Directed Mutagenesis Kit (Stratagene) according to their protocol. In addition, a construct was made in which the carboxy-terminus was truncated by introducing a stop codon after Pro 245. All mutations were confirmed by sequencing and the construct was subsequently used to transform *Pichia pastoris* X-33 cells. High yielding transformants were selected based on growth on high Zeocin concentrations as described previously [25].

### Protein production and purification

Clones were grown in 3L fermentors with glycerol and methanol feed phases. Harvested cells were resuspended in breaking buffer (50 mM phosphate buffer pH7.5, 5% glycerol) and broken by three passages through an X-press cell (Biox AB). Cell debris was pelleted by centrifugation at 10,000 × g for 30minutes. This centrifugation step was repeated to ensure that all debris had been removed. Membranes were collected at 200,000 × g for 90 minutes, washed in urea buffer (4M urea, 5 mM Tris, 2 mM EDTA 2 mM EGTA) and NaOH (20 mM) and finally resuspended in 7 ml resuspension buffer (20 mM Hepes pH7.8, 50 mM NaCl, 10% glycerol, 2 mM β-MeOH) per gram of membrane.

Membranes were solubilized in resuspension buffer with 6% n-nonyl-β-D-glucoside for 1h at room temperature. Unsolubilized material was removed by centrifugation at 186,000g for 30min. The supernatant was diluted with dilution buffer (20 mM MES pH6.0, 10% glycerol, 0.4% NG and 2 mM β-MeOH) and purified using a Resource S column (GE Healthcare, 20 mM MES pH6.0, 0.4% NG and 15 mM-1M NaCl) followed by gel filtration on Superdex 200 column (GE Healthcare, 20 mM Tris-HCl pH7.5, 100 mM NaCl, 0.4% NG). Fractions containing hAQP5 S156E was concentrated in a 10,000 MWCO concentrator (Vivaspin) to a final concentration of 7–13 mg/ml.

### Crystallization

Crystallization experiments were set up at 8°C using the hanging drop vapor diffusion technique. 7.2 μl reservoir solution (100 mM Tris-HCl pH7.8 or 7.9, 100 mM NaCl, 21% PEG400) was mixed with 1.8 μl each of 1,6-hexanediol (30%, v/v) and 1,3-propanediol (40%, v/v) and mixed with the protein in a 1:1 ratio. Crystals were obtained within 5–7 days and were fished and flash frozen in liquid nitrogen without any addition of cryoprotectant.

### Data collection and structure determination of AQP5 S156E

Complete X-ray diffraction data were collected on frozen crystals at cryo temperature (100 K) at the European Synchrotron Radiation Facility (ESRF) beamline ID23-2. Crystals of full-length AQP5 S156E diffracted to 3.5 Å and belonged to space group P3_1_2 with one tetramer in the asymmetric unit. Cell dimensions were a = b = 174.3, c = 100.9 Å, A = B = 90°, γ = 120°. Crystals of truncated AQP5 S156E crystals diffracted to 2.6Å and belonged to the space group P6_3_ with two tetramers in the asymmetric unit. Cell dimensions were a = b = 171 Å, c = 171, 190 Å, A = B = γ = 90°. Images were processed and scaled using iMosflm and Scala from the CCP4 suite [26]. The structures was solved using molecular replacement using Phaser [27] with the wild-type hAQP5 structure (PDB code 3D9S [23]) as a model. Further refinement of full-length and truncated AQP5 S156E was done in Refmac5 [28] and Phenix [29] respectively, with iterative manual rebuilding in Coot [30]. The quality of the structure was checked using MolProbity included in Phenix. The current model of full length AQP5 S156S contains 4 chains (A-D) with the following amino acids: A 2–245, B 5–247, C 4–245 and D 2–254. A-D as well as one phosphatidylserine. The current model of truncated hAQP5-S156E contains 8 chains A-H with amino acids 2–245, one phosphatidyl serine and 690 waters. The two structures refined to an R_work_ and R_free_ of 28.0 and 31.1% for full length AQP5 S156E and 18.7% and 23.0% for truncated AQP5 S156E. For crystallographic statistics, see Table 1.

**Table 1:** Crystallographic data and refinement statistics for full-length and truncated hAQP5 S156E structures.

	Full length	Truncated
*Data collection*		
Beamline	ESRF ID23-2	ESRF ID23-2
Wavelength (Å)	0.8726	0.8726
Detector distance (mm)	413	306.6
Oscillation/collection range (°)	1/100	0.4 (92)
Resolution^a^ (Å)	101.02–3.5 (3.69-3-5)	50.81–2.60 (2.74–2.60)
Total observations^a^	142,487 (20,652)	375,143 (54,612)
Unique reflections^a^	21,914 (3,193)	94,885 (13,925)
Completeness^a^	98.9 (99.8)	98.8 (99.4)
Redundancy^a^	6.5 (6.5)	4.0 (3.9)
I/σ^a^	13 (1.9)	7.0 (2.1)
R_sym_ ^a^ ^,^ ^b^ (%)	0.095 (0.799)	0.180 (0.778)
*Refinement*		
Resolution^a^ (Å)	100–3.5	48.2–2.60
Space group (α, β, γ)	P3_1_2 (90, 90, 120)	P6_3_ (90, 90, 120)
Cell dimensions (*a*, *b*, *c*)	174, 174, 101	171, 171, 190
Molecules in the asymmetric unit	4	8
R_work_ ^c^/R_free_ ^d^ (%)	28.0/31.1	18.7/23.0
Average B valules, entire model (Å^2^)	161.6	31.4
Average B values, protein/waters (Å^2^)	163.4/-	31.1/35.6
*Root-mean-square deviations from ideal values*		
Bond lengths (Å)	0.007	0.008
Bond angles (°)	1.084	1.058
PDB accession code	5DYE	5C5X

^a^ Values in brackets are for the highest resolution shell

^b^ R_sym_ = Σ_h_Σ_i_|*I*_i_(h)-<*I*(h)>|/ Σ_h_Σ_i_I(h), where *I*_i_(h) is the *i*th measurement.

^c^ R_work_ = Σ_h_||*F*(h)_obs_|-|*F*(h)_calc_||/Σ_h_|*F*(h)_obs_|

^d^ R_free_ was calculated for 5% of reflections randomly excluded from the refinement

## Results

### A phosphomimetic mutation at Ser 156 increases constitutive AQP5 surface abundance

**Fig 1:**
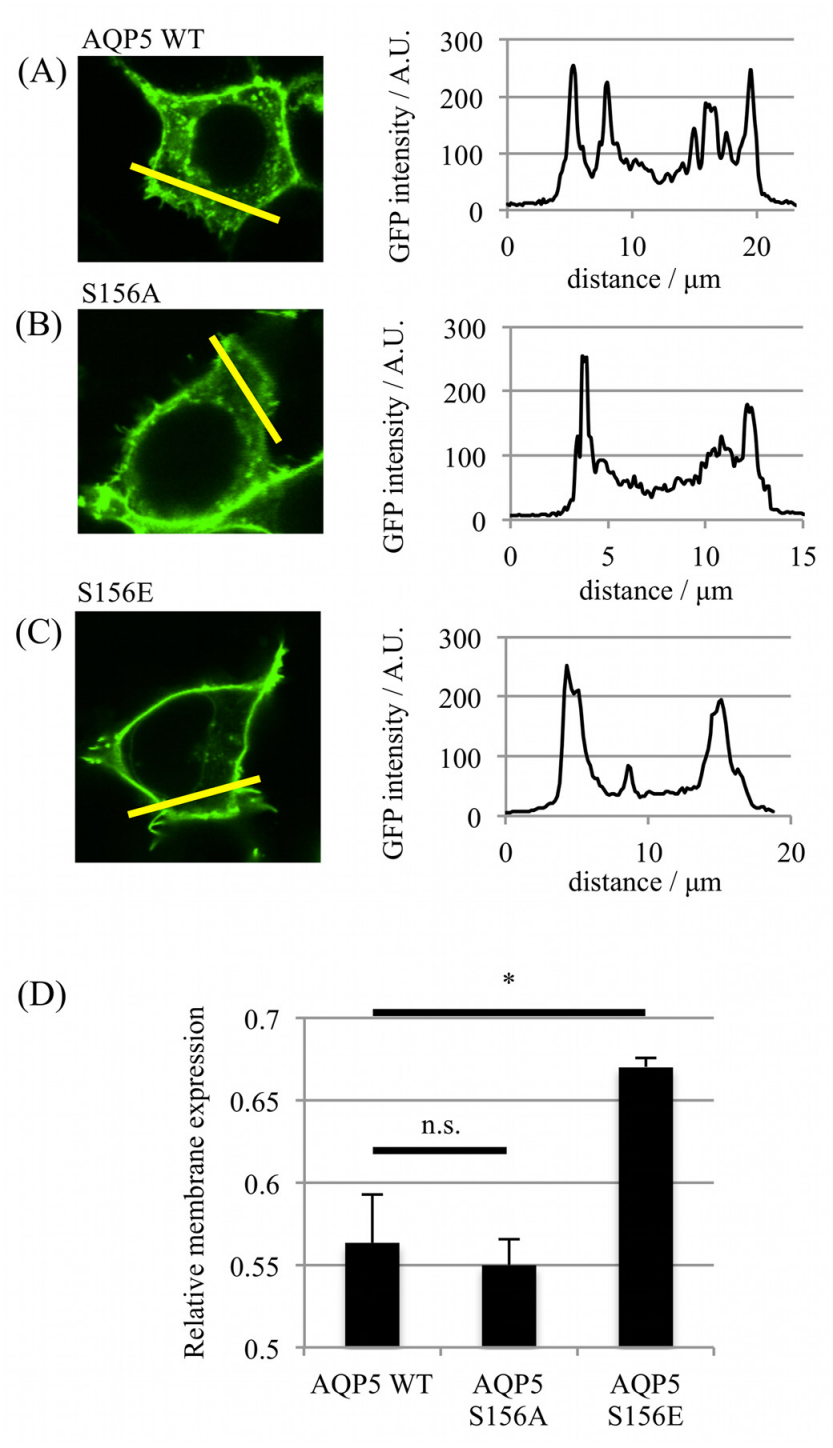
**Surface expression of AQP5 S156 mutants.** Representative fluorescence confocal micrographs of HEK293 cells transfected with GFP-tagged (A) AQP5 wild-type, (B) AQP5-S156A and (C) AQP5-S156E; the fluorescence intensity profiles along each yellow line are shown. (D) Relative membrane expression calculated from fluorescence intensity profiles: 5 profiles were taken per cell and at least 3 cells per micrograph, repeated in 3 independent experiments. Asterisks denote p < 0.05 using Student’s t-test followed by Bonferroni correction for multiple comparisons.

An expression vector was made in which GFP was fused to the carboxy-terminus of full-length human AQP5 (AQP5-GFP). This expression vector was used to transfect live HEK293 cells. Real-time localization and translocation of the corresponding fusion protein was measured using confocal microscopy; the relative membrane expression (RME) was determined using line intensity profiles generated from confocal images of individual live cells. An RME of 0 corresponds to an equal distribution of GFP signal between membrane and intracellular compartments and an RME of 1 corresponds to 100% of the GFP signal at the membrane. The RME of AQP5-GFP was increased by the introduction of the S156E mutation from 0.56 ± 0.02 to 0.67 ± 0.01 (p = 0.01), whereas the RME of AQP5-S156A was not significantly different (p = 0.36) to wild-type AQP5-GFP (Fig 1).

### PKA inhibition increases constitutive AQP5 surface abundance

**Fig 2:**
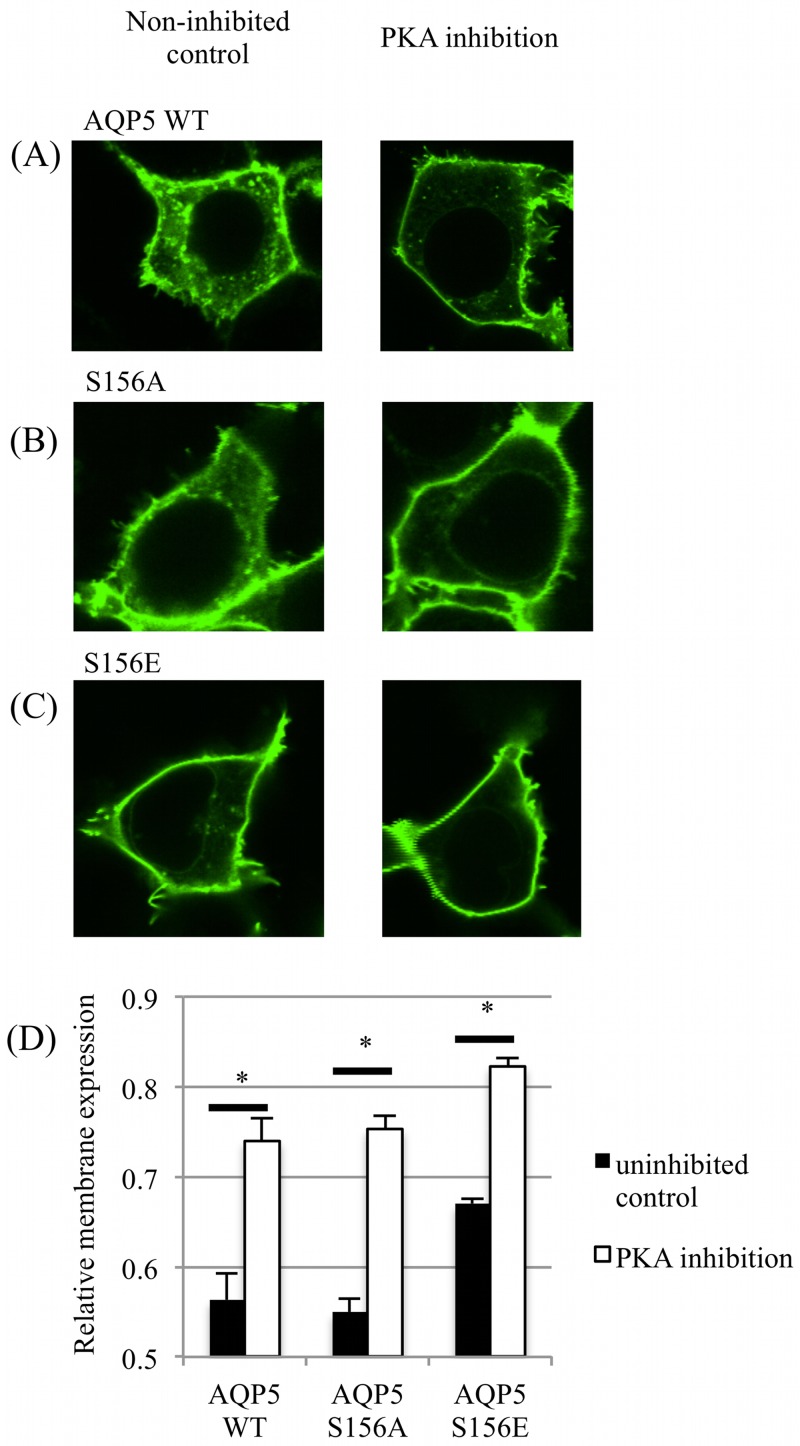
Effect of PKA inhibition on surface expression of AQP5. Representative fluorescence confocal micrographs of HEK293 cells transfected with GFP-tagged (A) AQP5 wild-type, (B) AQP5-S156A and (C) AQP5-S156E that were treated with a PKA inhibitory peptide for 30 minutes. (D) Relative membrane expression of the 3 AQP5 constructs with and without PKA inhibition. Asterisks denote p < 0.05 using Student’s t-test followed by Bonferroni correction for multiple comparisons.

The RME of AQP5-GFP (wild-type and the two S156 mutants) was measured after a 30 minute incubation with a cell-permeable PKA inhibitory peptide (myrPKI) or vehicle control. PKA inhibition increased the RME of all AQP5 constructs (Fig 2) compared to non-inhibited controls, suggesting a role for PKA signalling in the internalization of AQP5 as previously suggested [15]. PKA inhibition further increased the membrane abundance of the S156E mutant, indicating that this effect is independent of the phosphorylation status of Ser 156.

### Hypotonicity-induced AQP5 translocation is independent of PKA activity and phosphorylation at S156

**Fig 3:**
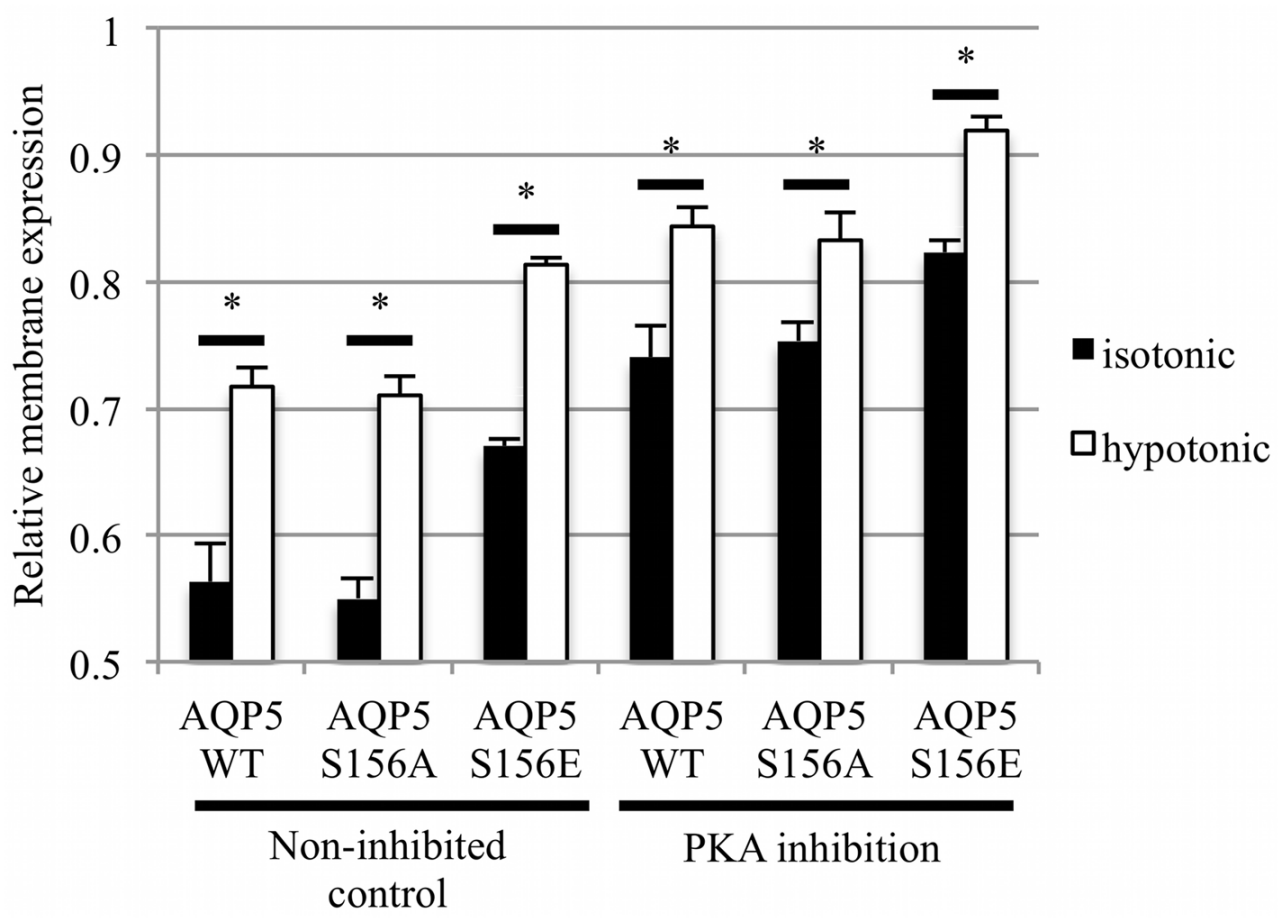
Hypotonicity-induced translocation of AQP5. Relative membrane expression of AQP5 and mutants was measured in the same cells before and 1 minute after reduction of the extracellular osmolality to 85 mOsm/kg H_2_O by fourfold dilution of the culture medium with dH_2_O. PKA inhibition was achieved by a 30 minute incubation with a PKA inhibitory peptide. Asterisks denote p < 0.05 by paired t-tests followed by Bonferroni correction for multiple comparisons.

Reduction of the extracellular osmolality to 85 mOsm/kg H_2_O caused rapid relocalization of AQP5 from intracellular compartments to the plasma membrane (Fig 3). The initial AQP5-GFP distribution profile was restored on returning to normal physiological osmolality. The RME of AQP5-GFP increased from 0.56 ± 0.03 in control medium to 0.72 ± 0.01 in hypotonic medium. This result was similar to that seen for AQP1, which translocated to and from the membrane in response to altered tonicity in the surrounding media [24]. Similarly, the RME increased from 0.55 ± 0.02 to 0.71 ± 0.02 for the S156A mutant and from 0.67 ± 0.02 to 0.81 ± 0.01 for the S156E mutant. Despite an increased level of constitutive surface expression, the change in RME for the S156E mutant (ΔRME = 0.14 ± 0.01) was similar to that for AQP5 or the S156A mutant (ΔRME = 0.15 ± 0.03 and 0.13 ± 0.02 respectively). Inhibition of PKA, despite increasing constitutive surface expression of all constructs, did not inhibit the hypotonicity-induced relocalization of AQP5. Taken together, these results suggest that hypotonicity-induced relocalization of AQP5 is not mediated by either PKA activity or phosphorylation at Ser 156.

### The crystal structure of AQP5 S156E suggests phosphorylation is not accompanied by a conformational change

We previously solved the structure of wild-type human AQP5 expressed in *P*. *pastoris* to 2.0 Å resolution [23]. To determine whether phosphorylation of Ser 156 confers any structural changes that could be important for membrane translocation, we crystallized and solved the structure of a single phosphomimetic mutant (S156E) of full-length AQP5 at 3.5 Å resolution (PDB code 5DYE). Since at this resolution, information about side-chain positions and interactions is limited, we truncated AQP5 S156E at Pro 245, the last visible residue in the high-resolution wild-type structure AQP5. The truncated AQP5 S156E construct crystallized in a different space group and diffracted to significantly higher resolution, allowing us to solve the structure at 2.6 Å resolution (PDB code 5CX6). Both full-length and truncated AQP5 S156E crystallized in space groups lacking the four-fold symmetry otherwise commonly associated with AQP crystals (P3_1_2 with one tetramer in the asymmetric unit for full-length AQP5 S156E and P6_3_ with two tetramers in the asymmetric unit for truncated AQP5 S156E), thereby allowing for structural differences between monomers in the tetramer to be examined. Crystallographic data and refinement statistics are summarized in Table 1.

**Fig 4:**
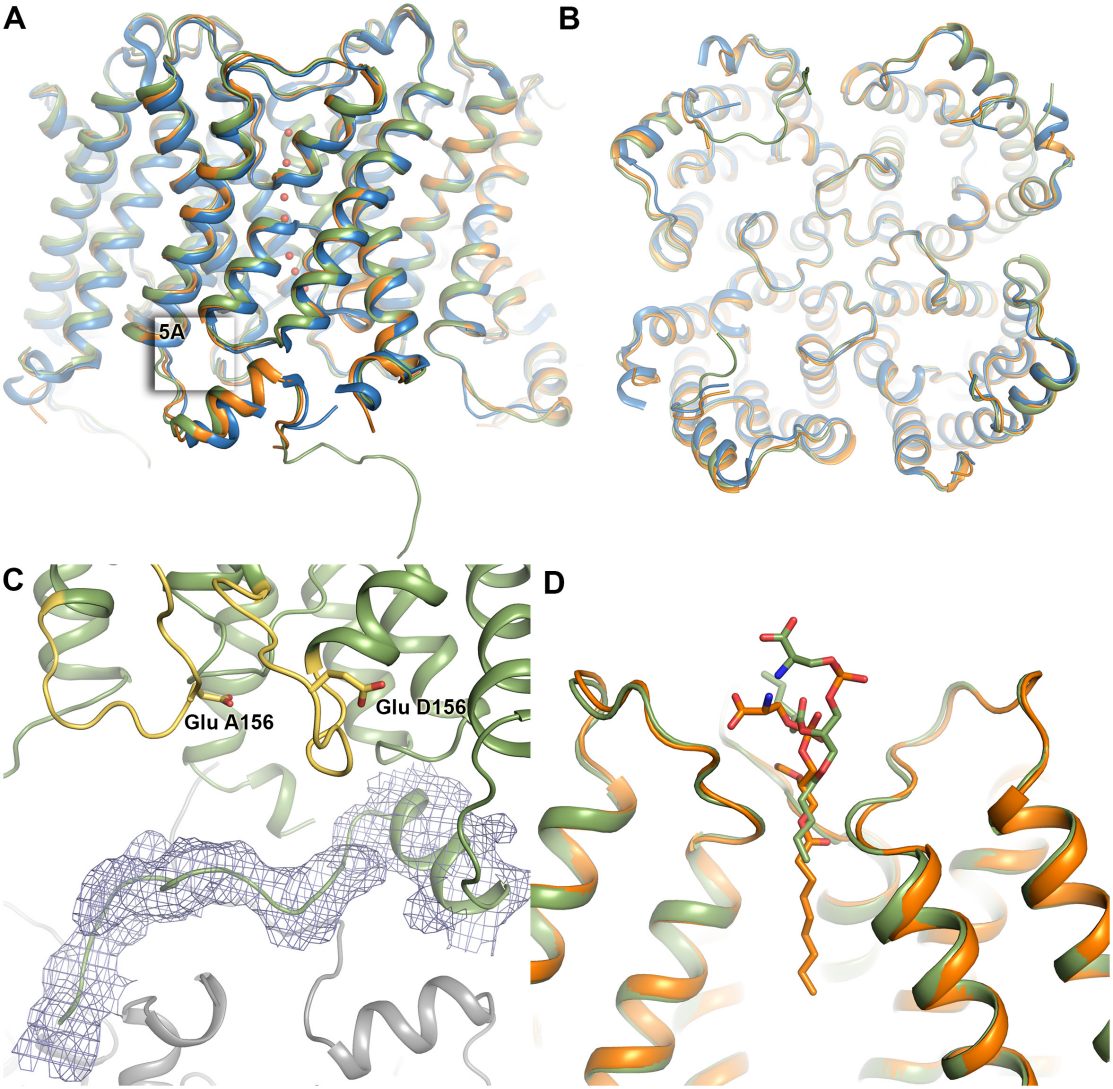
**Overall structure of AQP5 S156E.** (A) The structure of full length (green, PDB code 4DYE) and truncated S156E AQP5 (orange, PDB code 5CX6) overlaid on the wild-type structure of AQP5 in blue (PDB code 3D9S). Water molecules in the water-conducting channel of truncated S156E AQP5 are shown as red spheres. (B) Same as in (A), viewed from the cytoplasmic side. (C) Structure of the carboxy-terminus of full-length S156E monomer D, showing its interactions with a symmetry-related molecule (grey). 2F_obs_-F_calc_ electron density is displayed at 1.0 σ. Loop D and Glu 156 in monomers A and D are highlighted in yellow. (D) Lipid molecule in the tetrameric channel of full-length and truncated AQP5 S156E.

Both structures of AQP5 S156E are very similar to that of wild-type AQP5; overlaying with a root-mean-square deviation of 0.51 Å for 970 C_α_-atoms for full-length AQP5 S156E and of 0.37Å for 975 C_α_-atoms for truncated AQP5 S156E (Fig 4A & 4B). For full-length AQP5 S156E, additional electron density could be seen beyond Pro 245 in one of the monomers (monomer D), allowing us to build nine more residues (Fig 4C). However, this region does not interact with any other residues within the tetramer. Instead, it forms crystal contacts with symmetry-related AQP5 molecules in the crystal packing, explaining why this region is ordered in this particular monomer of the full-length structure.

In the truncated AQP5 S156E structure, seven water molecules were observed in the water-conducting channel of all monomers with one exception, monomer F, which contained six water molecules. For full-length AQP5 S156E, the limited resolution prevented the identification of water molecules in the channel. In both structures, a lipid molecule could be observed in the central channel formed between monomers in the tetramer, albeit at slightly different positions. Similarly to wild-type AQP5, this lipid was modelled as phosphatidyl serine (Fig 4D).

### Loop D and the carboxy-terminus retain their native conformations in AQP5 S156E

**Fig 5:**
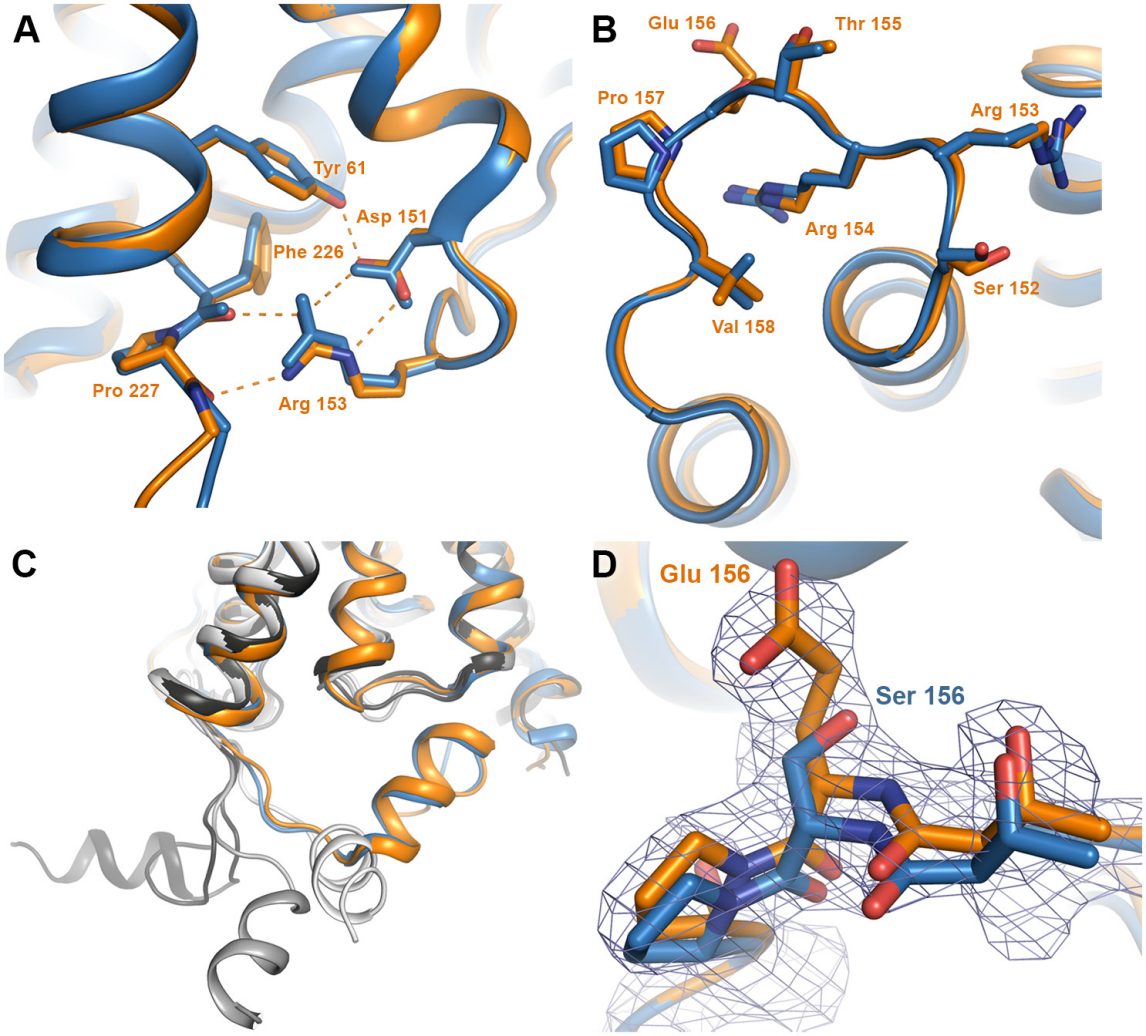
Structure of loop D and its interaction with the carboxy-terminus. Wild-type AQP5 and AQP5 S156E are coloured blue and orange respectively. (A) Zoomed in view of the boxed area in Fig 4A showing that the interactions between loop D and the carboxy-terminus are maintained in the S156E mutant structure. Hydrogen bonds are shown as dashed lines. Structural comparison of (B) loop D and (C) the carboxy-terminus shows that there are no structural differences between wild-type AQP5 and AQP5 S156E. In (D), the four monomers from the crystal structure of human AQP2 are overlaid, showing a significant conformational variability of the carboxy-terminal helix within the tetramer. The four AQP2 monomers are colored in different shades of grey. (D) Structure of the S156E mutation site showing 2F_obs_-F_calc_ electron density contoured at 1.0 σ. The structure of wild-type AQP5 is shown in blue for comparison.

In the wild-type AQP5 structure, an interaction between loop D and the carboxy-terminal region was observed [23]. This interaction anchored at the carboxy-terminal helix in a manner that was well conserved in other mammalian AQP structures and involved hydrogen bonds between the sidechain of Arg 153 and the backbone atoms of Pro 226 and Phe 227 (Fig 5A). It was hypothesized that phosphorylation of Ser 156 may cause structural changes within loop D that breaks these interactions, allowing for a conformational change of the carboxy-terminus.

Since there were no significant differences between the strucutres of full-length and truncated AQP5 S156E, we used the higher-resolution truncated AQP5 S156E structure to examine the finer structural details (from hereon denoted as AQP5 S156E). No structural change of loop D was observed in any of the eight monomers when compared to wild-type hAQP5 (Fig 5B) and the interactions between loop D and the carboxy-terminus were conserved (Fig 5A). The carboxy-terminus retained the same conformation as in the wild-type AQP5 structure (Fig 5C). In two of the monomers in each tetramer, there was a 1–2 Å shift in the position of the small carboxy-terminal helix when compared to wild-type AQP5. Comparison between the 8 monomers of AQP5 S156E shows that the carboxy-terminal helices overlap perfectly, suggesting that the shift in position arises from minor structural differences between monomers in wild-type AQP5. As is apparent from the electron density map in Fig 5D, serine was successfully replaced by glutamate at position 156.

## Discussion

The regulation of protein abundance in a particular membrane requires a delicate balance between two opposing processes: Translocation to and internalization from the membrane. This is achieved through multiple sorting signals, often consisting of post-translational modifications in the cytoplasmic regions of the protein. One of the best-characterized examples is AQP2, for which phosphorylation of multiple sites in the carboxy-terminus governs its translocation to the apical membrane [31]. Internalization of AQP2 is triggered by ubiquitination of the carboxy-terminus [32] and the internalization rate is also influenced by the phosphorylation status of AQP2 in the membrane [33].

Emerging evidence suggests that the regulation of AQP5 cell surface abundance is equally complex. It is known that AQP5 translocation between intracellular storage vesicles and the apical membrane can occur upon agonist stimulation of the muscarinic (M3) receptor [34] or α-1 adrenergic receptor [35]. Furthermore, intracellular cAMP levels have been shown to regulate AQP5 abundance at the transcriptional level as well as affect the long-term (≥ 24 hours) sub-cellular distribution of the protein in a PKA-dependent manner [11]. Interestingly, increased cAMP levels have been shown to have a biphasic effect on the sub-cellular distribution of AQP5 with decreased expression in the apical membrane in the short term due to increased internalization, followed by an increase in membrane abundance in the long-term [15]. While inhibition of PKA had an effect on both components of this biphasic response, an increase in levels of phosphorylated AQP5 could only be seen for the long-term effect, suggesting that the target for PKA-phosphorylation in the short-term response is not AQP5 itself. This dual response to increased levels of cAMP on the trafficking of AQP5 could help explain why increased as well decreased expression in the apical membrane have been reported [14, 16].

AQP5 contains two consensus PKA-sites but unlike AQP2, their roles in AQP5 translocation have not been determined. PKA-mediated phosphorylation of AQP5 has been demonstrated at both Ser 156 [21] and Thr 259 [36]. However, removal of these phosphorylation sites by mutation to alanine resulted in constructs with the same membrane abundance as wild-type AQP5. Additional phosphorylation sites are predicted to be present in AQP5, for example a PKC site at Ser 152 that overlaps with the PKA site at Ser 156. Furthermore, Thr 259 is also part of a PKG consensus site. These are additional components that may affect the overall translocation of AQP5 in a cell-dependent manner.

**Fig 6:**
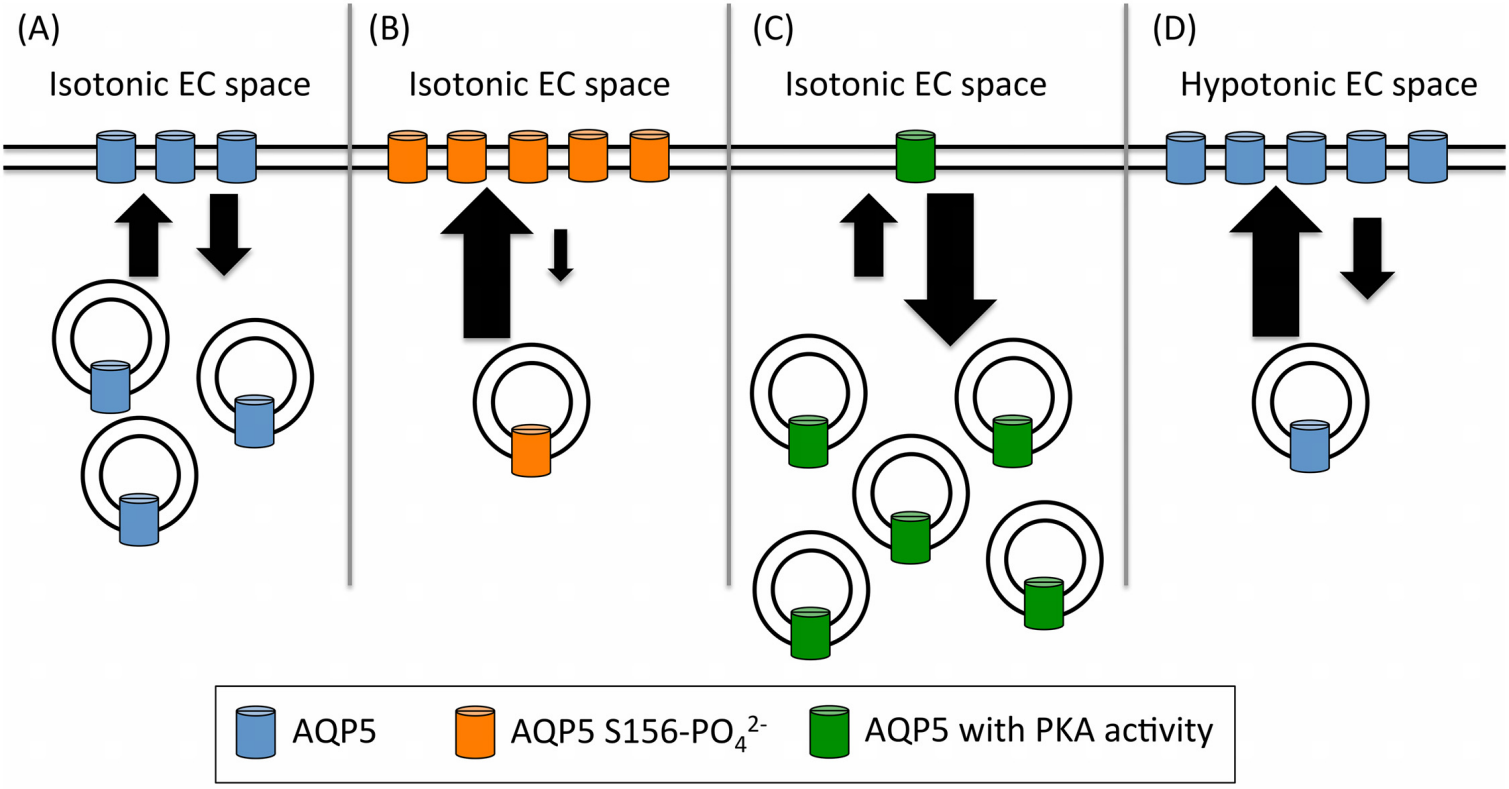
A proposed model of the equilibrium between vesicular and surface-localized AQP5. Panel A shows wild-type AQP5 under isotonic conditions. The large arrows represent an increase in AQP5 translocation (and the small arrows are a decrease). This is regulated by three independent factors: Phosphorylation of AQP5 at position S156 (orange cylinders in panel B; shown by a phosphomimetic glutamate substitution (S156E) of AQP5; the effect of PKA (green cylinders in panel C) and the effect of decreasing the relative tonicity of the environment (panel D). We speculate that these three pathways control the surface abundance of AQP5.

Our data suggest that membrane-localization of AQP5 is regulated by at least three independent pathways (Fig 6). We show that membrane expression of AQP5 is affected by phosphorylation of Ser 156, either by increased targeting or decreased internalization or both. To our knowledge this is the first time a phosphorylation site has been directly linked to a difference in membrane expression. The fact that the S156A mutant behaves like wild-type AQP5 indicates that phosphorylation of Ser 156 may not occur under basal conditions. In this respect it is intriguing that Ser 156 has been shown to be preferentially phosphorylated in tumour cells [21]. It has been suggested that in tumours, PKA-dependent phosphorylation of Ser 156 increases cell proliferation by activating the Ras-pathway [37]. A number of studies have shown that upregulation of AQPs promotes cell proliferation and migration [38] Our data hint at a mechanism whereby phosphorylation of Ser 156 in AQP5 increases its membrane localization, thereby enhancing cancer cell proliferation.

It was previously suggested that phosphorylation of Ser156 would break interactions between loop D and the carboxy-terminus, thereby triggering a conformational change [23]. Significant conformational variability of the carboxy-terminus was seen in the structure of human AQP2, where the carboxy-terminal helix of each monomer adopted a unique conformation that had not been observed in any previous mammalian AQP structure (Fig 5C) [39]. As the carboxy-terminal region is known to be important for membrane translocation of both AQP2 [31] and AQP5 [10, 40], such conformational changes may be recognized as a structural sorting signal by the cellular trafficking machinery. However, our structure of the AQP5 S156E mutant did not reveal any structural differences within loop D or the carboxy-terminus (Fig 5B and 5C). We therefore suggest that it is the presence of a phosphate group at Ser 156 rather than a conformational change of the carboxy-terminus that is recognized as a sorting signal, resulting in increased AQP5 membrane abundance.

Our data further show that, independent of Ser 156, basal levels of PKA activity (i.e. in the absence of a cAMP-stimulating agonist) decrease the membrane abundance of AQP5 (Fig 2). This fits well with the short-term effect of cAMP and PKA that has been seen previously and for which PKA has been suggested to be involved in AQP5 internalization [15]. Since PKA-inhibition further increased membrane expression of the S156E mutant, this indicates that phosphorylation of Ser 156 is not responsible for this short-term effect. Instead, we suggest that Ser 156 phosphorylation may be involved in the long-term cAMP and PKA-dependent effect whereby an increase in AQP5 membrane abundance is seen after several hours. This is in agreement with the observation that the long-term effect of cAMP and PKA involves direct phosphorylation of AQP5 while the short-term effect does not [15].

Finally, we show that AQP5-GFP is rapidly translocated to the target membrane of HEK293 cells in response to hypotonic conditions, but that this translocation is independent of Ser 156 in loop D and also independent of PKA (Fig 3).

The hypotonicity-induced membrane translocation of AQP5 described here is consistent with that of AQP1 where the phosphorylation of Thr157 and/or Thr239 by PKC results in rapid accumulation in the cell-surface membrane [13]. Whether direct phosphorylation of AQP5 by PKC or another kinase mediates hypotonicity-induced membrane translocation in a similar manner remains to be shown. AQP5 is found in tissues that are subject to rapid changes in osmolarity and has been shown to play an important role in cell volume regulation [14–16]. Upon exposure to hypotonic conditions, cells undergo a rapid cellular volume decrease (RVD) to avoid rupture. This happens within a couple of minutes and involves the release of cellular water. RVD is often followed by a regulatory volume increase (RVI) whereby the osmotically shrunken cell approaches its original volume [2]. The rapid response to hypotonicity reported here supports the involvement of AQP5 in RVD as previously suggested [14]. On a longer time scale, hypotonic exposure has been shown to cause a decrease in AQP5 membrane abundance [16], most likely corresponding to the RVI response. Taken together, regulation of AQP5 membrane abundance seems to be important for both the RVD and RVI components of cell volume regulation.

We previously showed that hypotonicity-induced calcium influx through TRPC1 was vital for translocation of AQP1 [13] and others have shown that calcium influx through TRPV4 mediates the AQP5-dependent regulatory volume decrease in acinar cells [14]. We therefore speculate that the sensor of extracellular osmolality that leads to AQP5 translocation is a member of the TRP family of gated cation channels.

## Conclusion

Due to its importance for fluid secretion in airways submucosal glands, AQP5 has been suggested to be a pharmacological target to treat the hyper-viscous and excessive gland secretions in cystic fibrosis and bronchitis/rhinitis, respectively. Our data provide the first link between a specific AQP5 phosphorylation site, Ser 156, and changes in its sub-cellular localization. We further show that the Ser 156-mediated change in localization does not require a substantial protein conformational change. We show that, independent of Ser 156, PKA is further involved in basal recycling of AQP5 between the plasma membrane and intracellular compartments. Finally, we provide evidence of tonicity-regulated changes in AQP5 localization that are not mediated by phosphorylation of Ser 156 or PKA. This will now enable us to elucidate the detailed mechanism by which these post-translational modifications govern translocation of the protein from intracellular storage vesicles to the target membrane in response to protein kinase activity and osmotic triggers, thus providing key information for drug design targeting AQP5.
